# Posterior Reversible Encephalopathy Syndrome as an Unusual Complication of Routine Chemotherapy: A Case Report

**DOI:** 10.7759/cureus.99045

**Published:** 2025-12-12

**Authors:** Ahmed Osman, Maximilian Bonnici, Samruddhi Jain, Douglas Burd

**Affiliations:** 1 Radiology, St. Vincent Hospital, Worcester, USA

**Keywords:** chemotherapy, encephalopathy, gemcitabine, mri, neurology, oncology, posterior reversible encephalopathy syndrome, radiology, vasogenic edema

## Abstract

Posterior reversible encephalopathy syndrome (PRES) is a rare and poorly understood clinico-radiological syndrome with the potential for devastating neurological symptomatology. There is increasing evidence that chemotherapy may contribute to the induction of PRES. Although poorly understood, it is theorized that gemcitabine leads to PRES by inducing vasogenic edema from endothelial dysfunction. We report a case of PRES in which a patient presented with seizures and autonomic instability after receiving gemcitabine. Magnetic resonance imaging (MRI) of the brain showed grey-white matter hyperintensities localized to the bilateral occipital lobes without evidence of hemorrhage or restricted diffusion. This case highlights the importance of early recognition of PRES, as timely management can prevent irreversible sequelae.

## Introduction

Posterior reversible encephalopathy syndrome (PRES) is a clinico-radiological syndrome that began being described at the end of the 20th century [1,2]. PRES involves a constellation of symptoms, including headaches, altered mental status, and seizures [1]. These symptoms can be non-specific, meaning a thorough clinical and radiological evaluation is optimal to clinch the diagnosis. PRES is typically associated with well-known risk factors, including eclampsia, renal insufficiency, acute rises in blood pressure, and collagen vascular diseases [2]. However, chemotherapeutics, especially tyrosine kinase inhibitors and platinum agents, can also increase the risk of PRES. Gemcitabine specifically is thought to lead to PRES via endothelial injury, vascular dysregulation, and metabolic derangements. Given that chemotherapy is suspected to induce PRES, clinicians should have a high index of suspicion of PRES in patients with acute neurological deterioration after being on such agents. This report details a case of PRES linked to gemcitabine therapy, underscoring the importance of recognizing this complication after receiving routine chemotherapy.

## Case presentation

A 75-year-old female patient with a known history of metastatic cholangiocarcinoma was admitted with complaints of anorexia and diffuse abdominal pain. She also had a reduced appetite one week before presenting to the emergency department. She had been diagnosed with stage IV cholangiocarcinoma in 2023 and was treated with a combination of gemcitabine, cisplatin, and durvalumab. At the time of admission, she was receiving maintenance therapy with gemcitabine and durvalumab.

Her abdominal pain was vague. She denied fever, nausea, vomiting, and diarrhea. Physical examination was remarkable for dry oral mucosa, icterus, and generalized abdominal tenderness on deep palpation.

Initial evaluation revealed obstructive jaundice with a total bilirubin level of 5.4 mg/dL and direct bilirubin of 4.2 mg/dL (Table 1). She was also noted to be anemic (hemoglobin 10.5 g/dL) and had leukocytosis (WBC 11.9×10^3^/mcL). Renal function, other liver enzymes, and serum biochemistry were within normal limits. Empiric antibiotic therapy was initiated for concern of sepsis secondary to acute cholangitis.

**Table 1:** Abnormal lab results on initial evaluation WBC: white blood cell; Hg: hemoglobin

Lab tests	Lab value	Reference range
WBC	11.9×10^3^/mcL	3.9-11.0×10^3^/mcL
Hg	10.5 g/dL	11.5-12.5 g/dL
Total bilirubin	5.4 mg/dL	0.1-1.2 mg/dL
Direct bilirubin	4.2 mg/dL	0-0.4 mg/dL

Abdominal imaging demonstrated significant intrahepatic and common bile duct dilation secondary to a malignant stricture. She subsequently underwent endoscopic retrograde cholangiopancreatography (ERCP) with sphincterotomy, sphincteroplasty, and placement of a covered stent.

Two days after ERCP, the patient experienced two episodes of new-onset generalized tonic-clonic seizures. The second seizure consisted of a shaking right upper limb. Both seizures were followed by significant post-ictal confusion. At the time of these events, she was afebrile but exhibited autonomic instability, with a heart rate fluctuating up to 160 bpm and labile systolic blood pressure ranging from 90 to 150 mmHg. Laboratory investigations were notable for a WBC of 11.7×10^3^/mcL, hemoglobin of 10.3 g/dL, hypokalemia with a potassium of 3 mEq/L (reference range of 3.6-5.6 mEq/L), and elevated serum lactate of 4.7 mmol/L (reference range of 0.5-1.9 mmol/L).

**Figure 1:**
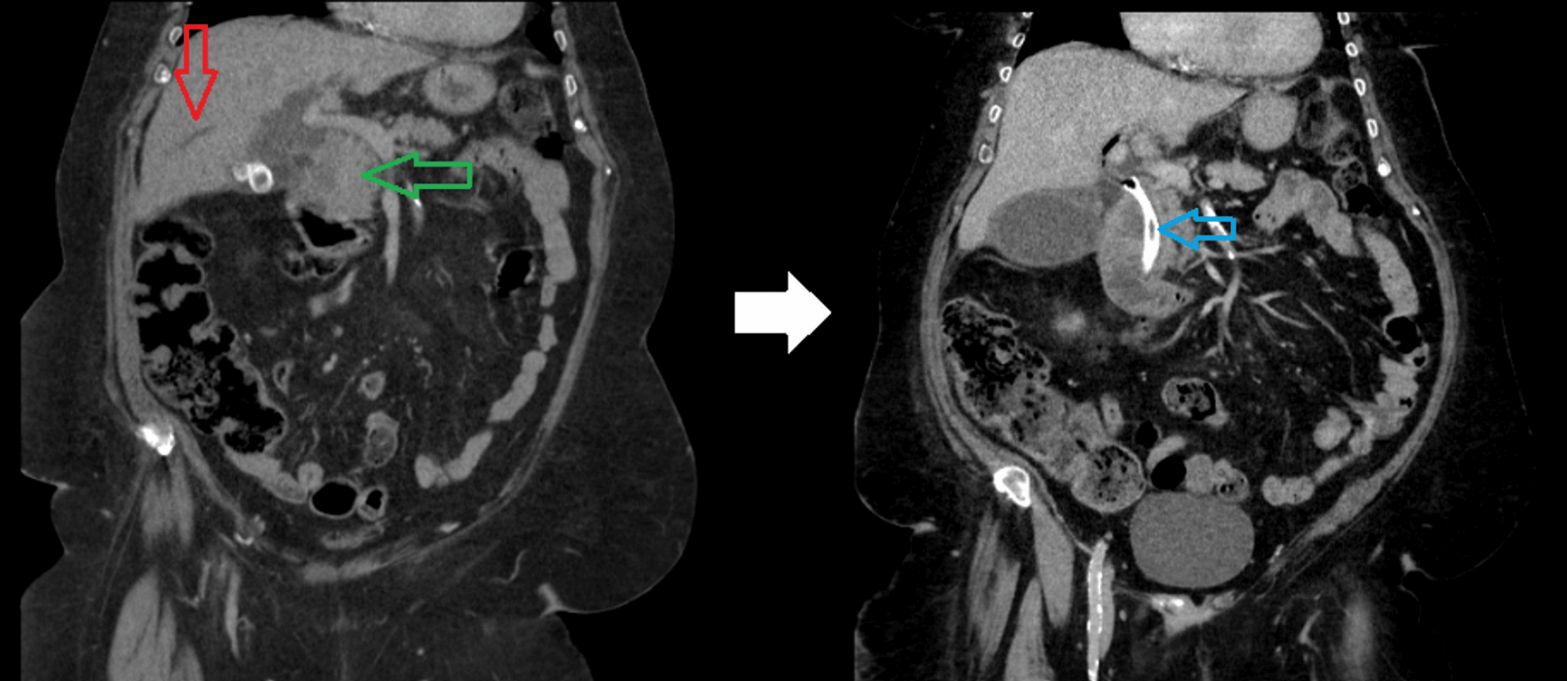
Cholangiocarcinoma imaging The left image shows a coronal view of a CT of the abdomen/pelvis. There is intrahepatic dilation (red arrow) as a result of an ampullary/pancreatic head lesion (green arrow). The image to the right of the white arrow is another coronal view of a CT of the abdomen/pelvis taken approximately a week later. It shows the placement of a common bile duct stent (blue arrow). CT: computed tomography

**Figure 2:**
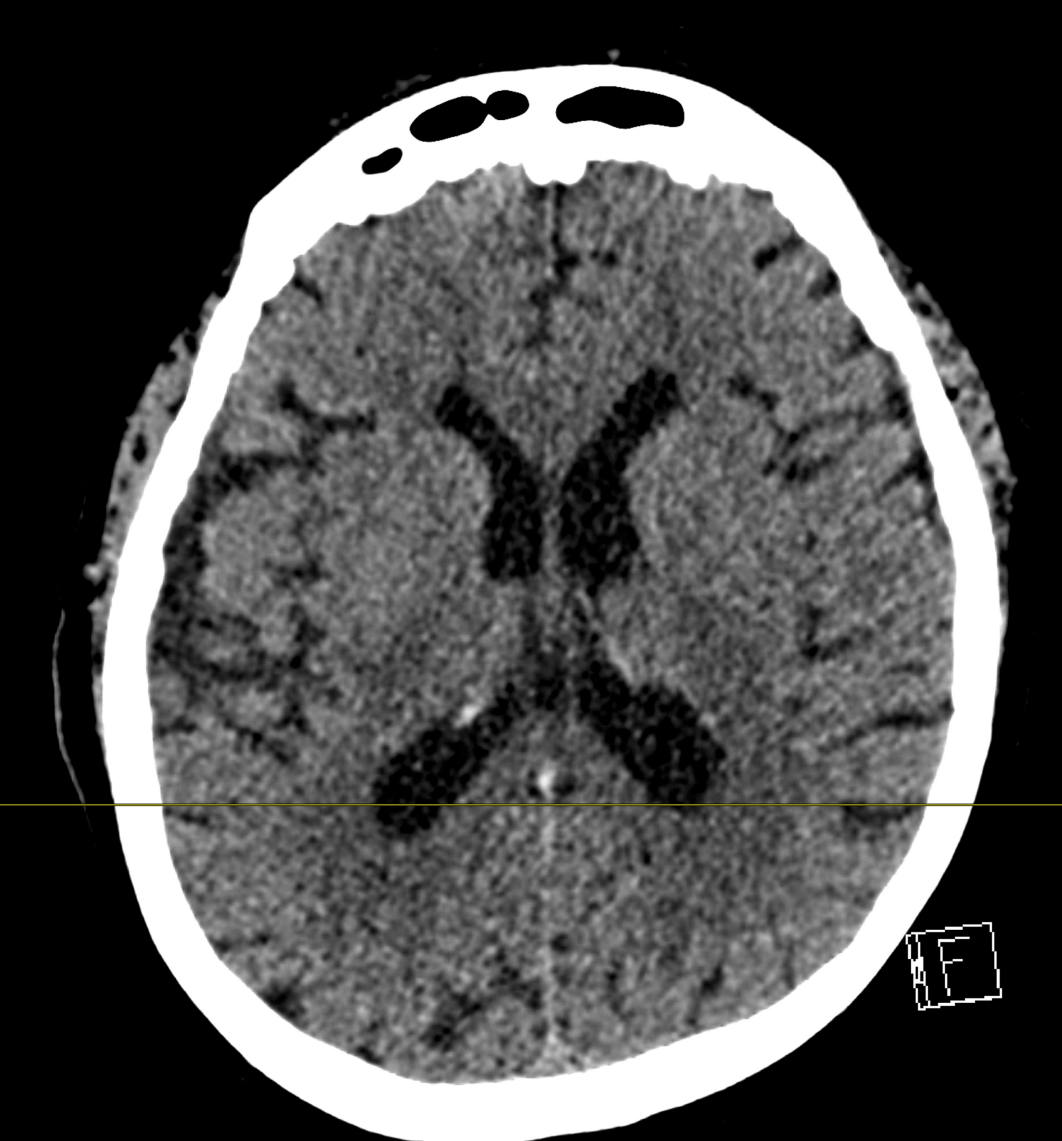
CT of the head Finding: Axial non-contrast CT of the brain showing normal age-appropriate prominence of ventricular system and cortical sulci without evidence of abnormality. Technique: Axial angled images, 120 kVp, 200 mA, 2 mm slice thickness. CT: computed tomography

**Figure 3:**
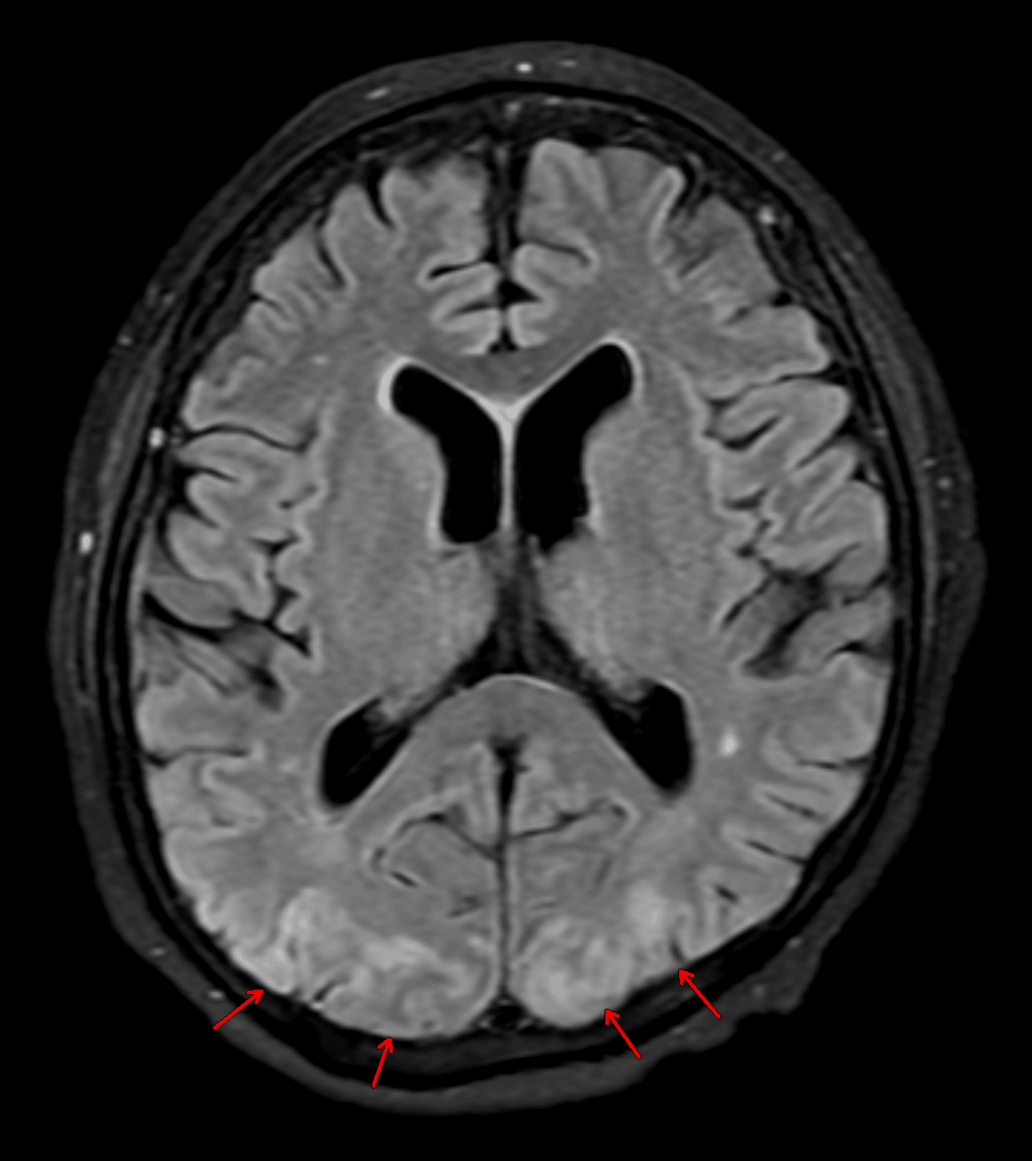
Hyperintensities on FLAIR Finding: Axial non-contrast FLAIR MRI brain showing symmetric hyperintensity (red arrow) in bilateral occipital lobes with effacement of adjacent cortical sulci. Technique: 1.5 T MRI, FLAIR sequence. FLAIR: fluid-attenuated inversion recovery; MRI: magnetic resonance imaging

**Figure 4:**
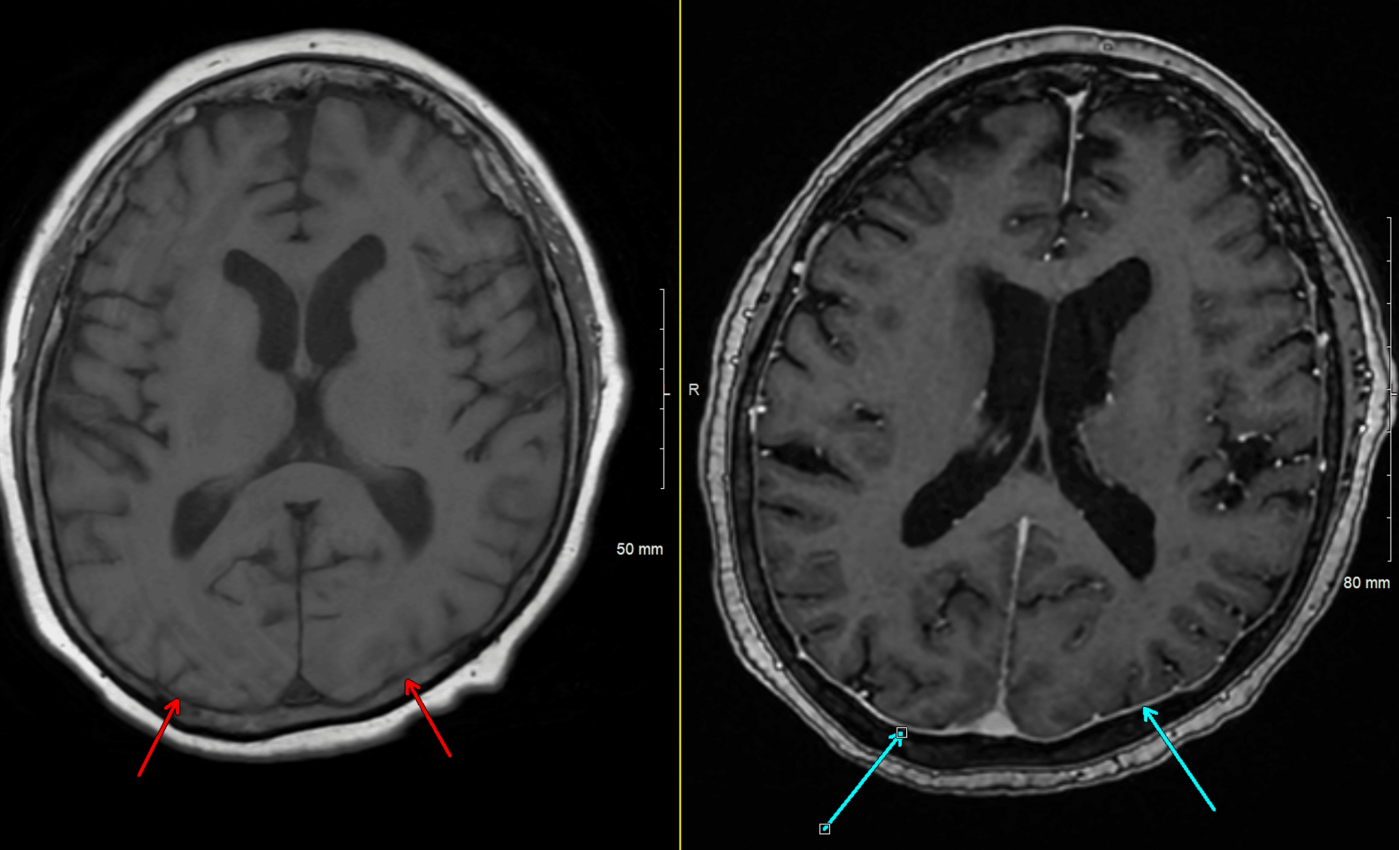
No abnormal enhancement Finding: Axial pre- (red arrow) and post-contrast T1 (blue arrow) MRI of the brain showing no abnormal enhancement in occipital lobes corresponding to the FLAIR hyperintensity. Technique: 1.5 T MRI, pre- and post-contrast T1 sequence. FLAIR: fluid-attenuated inversion recovery; MRI: magnetic resonance imaging

**Figure 5:**
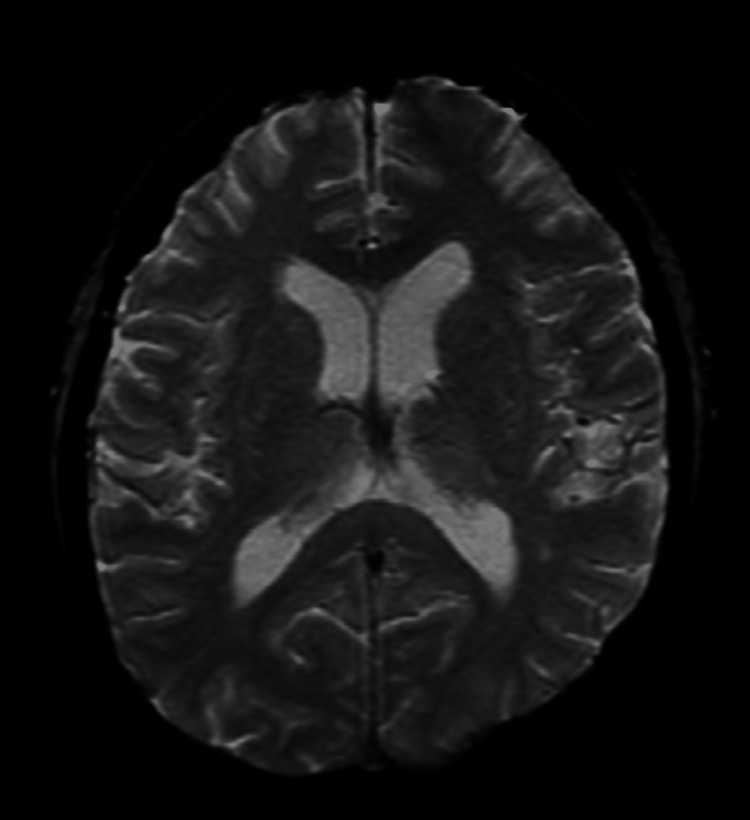
No evidence of hemorrhage Finding: Axial MRI of the brain GRE sequence showing no evidence of hemorrhage. Technique: 1.5 T MRI, GRE. MRI: magnetic resonance imaging; GRE: gradient-recalled echo

Neuroimaging was performed, including a non-contrast computed tomography (CT) (Figure 1) of the brain and a contrast-enhanced magnetic resonance imaging (MRI) of the brain. Brain MRI revealed hyperintensities involving white and grey matter bilaterally, predominantly affecting the parietal and occipital lobes on fluid-attenuated inversion recovery (FLAIR) sequence imaging (Figures 2-3), which is consistent with PRES. There was no associated diffusion restriction or hemorrhagic components (Figures 4-5).

The patient was suspected of having gemcitabine-induced PRES based on her clinical presentation and MRI findings. She then received appropriate electrolyte repletion and received lorazepam and a loading dose of levetiracetam. Subsequent improvement in heart rate was noted with persistently elevated systolic blood pressure up to 170 for the next two days before normalization. Her chemotherapy was discontinued due to acute illness, and it was decided to start FOLFOX (folinic acid, fluorouracil, and oxaliplatin) treatment on an outpatient basis. This was done to continue cholangiocarcinoma management while minimizing further PRES risk.

An electroencephalogram (EEG) was performed, which was unremarkable. The patient was started on levetiracetam maintenance therapy. This appeared to work well as the patient did not have any further seizures or neurological deficits throughout her remaining week in the hospital. An outpatient brain MRI with and without IV contrast was recommended to be done one month after the patient's seizures to reassess for PRES improvement. However, after the patient's discharge, she was lost to follow-up.

## Discussion

PRES is increasingly recognized in various clinical contexts, yet its exact pathophysiological mechanism remains incompletely understood. The prevailing hypotheses involve endothelial dysfunction leading to impaired cerebral vascular autoregulation and subsequent vasogenic edema [1]. Several precipitating factors have been traditionally associated with PRES, most notably acute elevations in blood pressure, renal insufficiency, and systemic inflammatory states [2].

Gemcitabine, a pyrimidine nucleoside antimetabolite, exerts its antineoplastic effects by incorporating into DNA and inhibiting ribonucleotide reductase, thereby disrupting DNA synthesis. Cases of PRES have been reported in patients receiving gemcitabine, either as monotherapy or in combination with other chemotherapeutic or immunotherapeutic agents [3]. Although the precise mechanism linking gemcitabine to PRES is not fully elucidated, it is speculated that chemotherapy-induced endothelial injury, vascular dysregulation, and metabolic derangements may contribute to the development of cerebral edema [1]. These changes may impair the blood-brain barrier, leading to the characteristic radiographic findings.

Radiologically, PRES is characterized by symmetric vasogenic edema, predominantly involving the parietal and occipital lobes [4]. MRI typically reveals hyperintensities on T2-weighted and FLAIR sequences in the affected regions. Diffusion-weighted imaging usually shows no evidence of restricted diffusion, distinguishing vasogenic from cytotoxic edema. However, in a minority of cases (approximately 11-26%), cytotoxic edema with diffusion restriction indicative of infarction can occur. Hemorrhagic complications are also reported in about 15% of cases, often visible on susceptibility-weighted imaging [4].

Currently, there is no disease-specific treatment for PRES. Management primarily focuses on the identification and correction of the underlying cause, such as blood pressure control, discontinuation of offending agents, and supportive care [5]. If not promptly recognized and managed, PRES can progress to irreversible neurological damage, including infarction, hemorrhage, or chronic cognitive deficits [6]. Therefore, early recognition of clinical features, contributing factors, and characteristic imaging findings is critical for improving outcomes.

## Conclusions

PRES is characterized by vasogenic edema, typically involving the parieto-occipital regions of the cerebral hemispheres. Specifically, gemcitabine is believed to cause vasogenic edema through endothelial dysfunction, resulting in neurological symptoms. This case serves as a reminder to clinicians to consider PRES as a potential complication of gemcitabine and other routine chemotherapies. Recognizing precipitating factors beyond hypertension, such as renal dysfunction, cytotoxic agents, and immunotherapy, is crucial for early diagnosis and intervention. Such timely management can prevent irreversible neurological injury.
